# Systematic Observation: Relevance of This Approach in Preschool Executive Function Assessment and Association with Later Academic Skills

**DOI:** 10.3389/fpsyg.2017.02031

**Published:** 2017-12-01

**Authors:** Elena Escolano-Pérez, Maria Luisa Herrero-Nivela, Angel Blanco-Villaseñor, M. Teresa Anguera

**Affiliations:** ^1^Faculty of Education, University of Zaragoza, Zaragoza, Spain; ^2^Faculty of Psychology, University of Barcelona, Barcelona, Spain

**Keywords:** systematic observation, child development, executive functions, academic competences, preschoolers, generalizability

## Abstract

Executive functions (EFs) are high-level cognitive processes that allow us to coordinate our actions, thoughts, and emotions, enabling us to perform complex tasks. An increasing number of studies have highlighted the role of EFs in building a solid foundation for subsequent development and learning and shown that EFs are associated with good adjustment and academic skills. The main objective of this study was to analyze whether EF levels in 44 Spanish children in the last year of preschool were associated with levels of literacy and math skills the following year, that is, in the first year of compulsory education. We used a multi-method design, which consisted of systematic observation to observe preschool children during play and selective methodology to assess their reading, writing, and math skills in the first year of compulsory primary education. General linear modeling was used to estimate the percentage of variability in academic skills in the first year of primary school that was explained by preschool EF abilities. The results showed that preschool EF level, together with participants and the instrument used to assess academic skills, explained 99% of the variance of subsequent academic performance. Another objective was to determine whether our findings were generalizable to the reference population. To make this determination, we estimated the optimal sample size for assessing preschool EFs. To do this, we performed a generalizability analysis. The resulting generalizability coefficient showed that our sample of 44 students was sufficient for assessing preschool EFs. Therefore, our results are generalizable to the reference population. Our results are consistent with previous reports that preschool EF abilities may be associated with subsequent literacy and math skills. Early assessment of EFs may therefore contribute to identifying children who are likely to experience later learning difficulties and guide the design of suitable interventions for the optimization of EFs.

## Introduction

Although not generally compulsory, preschool is essential for early childhood development. This stage of education can determine children’s later development and learning and, consequently, performance and success at school and work, as well as in their personal and social lives (Duncan and Magnuson, 2013; Bartik, 2014). In these first years of life, the main neural connections that provide the basis for learning and behavior are established through the constant interaction of neurobiological factors and the stimulation of the child’s surroundings (Bick and Nelson, 2017). During preschool, it is possible to take early action to avoid or compensate for situations arising from personal, family, and/or social inequalities that can subsequently have an impact on development and learning throughout childhood and into adulthood (Kaufman et al., 2015).

After finishing preschool, children begin compulsory primary education. Primary school presents children with a context that is very different from preschool: teacher–student interaction is less emotional; greater autonomy is expected of students; the curriculum is more oriented toward reading, writing, and mathematics; work periods are longer and require more sustained attention and concentration, etc. Because of these new characteristics and expectations, for many children the transition to primary school is a stressful period characterized by excessive demands and various difficulties (Veličković, 2013; Harper, 2016). In fact, some children who adapt well to preschool experience a decrease in skill level when they start primary school: they become less active, more easily distracted, less eager to learn and participate in class activities, more dependent, more insecure, and have more problems in their peer relationships (Veličković, 2013). This academic and socioemotional maladjustment can contribute to the likelihood that children will become inactive students in primary school and can even harm their overall well-being by causing additional health and emotional problems. By contrast, children who adjust well to this transition are generally successful in primary school and also later in life (Veličković, 2013; Harper, 2016).

Recent studies in this area (Blair and Raver, 2015; Moriguchi et al., 2016) have found that preschool executive functions (EFs) are essential to building a solid foundation for subsequent development and learning and are associated with school adjustment and academic success at the start of primary education. Consequently, research on preschool EFs has increased considerably over the past decade. However, many aspects of preschool EFs—including how best to evaluate them—remain poorly understood. Preschool EFs are an area of study in which conceptual aspects are better understood than aspects related to development and measurement (Willoughby and Blair, 2016). In order to help overcome these limitations, this study provides an example of how systematic observation, applied in children’s natural context, can be an appropriate tool for assessing preschool EFs. On the basis of this assessment, we analyze the extent to which preschool EFs may be associated with academic skills 1 year later, in the first year of primary education. We also analyze whether the results obtained with the convenience sample recruited can be generalized to the reference population. Our use of generalizability theory (G theory) for this purpose represents a novel contribution in the measurement of preschool EFs in observational studies.

### Preschool Executive Functions

EFs are a family of high-level cognitive processes that allow for conscious, goal-directed control of thoughts and actions, making it possible to solve problems effectively and efficiently, particularly in novel situations (Diamond, 2013; Carlson et al., 2016; Zelazo et al., 2016). In the preschool years, EFs consist of three main processes: working memory, inhibition, and cognitive flexibility (or shifting or switching) (Miyake et al., 2000; Diamond, 2013; Howard et al., 2015; Carlson et al., 2016; Moriguchi et al., 2016).

Working memory is the ability to hold information active in one’s mind and mentally work with it for brief periods of time as a platform for guiding one’s behavior. Two types of working memory are distinguished by the content: verbal or semantic working memory, on the one hand, and non-verbal or visuospatial working memory, on the other (Miyake et al., 2000; Diamond, 2013).

Inhibition refers to the ability to control one’s behavior, thoughts, and/or attention in order to override a strong internal predisposition or external lure. It includes (a) behavioral inhibition (or inhibition of action) to control or cancel one’s motor behavior, resist temptations, and not act impulsively; (b) cognitive inhibition to control and/or tune out thoughts and memories; and (c) resistance to distractor interference (or inhibition of attention) to select the information or stimulus one needs to complete a task while ignoring competing distractions (Friedman and Miyake, 2004).

Cognitive flexibility is the ability to quickly adapt one’s course of thought or action to the changing demands of a situation. This involves being able to shift one’s attention from one condition of a task—e.g., stimulus, dimension, or rule—to another (Miyake et al., 2000; Diamond, 2013).

These three EFs undergo considerable development during the preschool years (Anderson and Reidy, 2012; Howard et al., 2015; Nieto et al., 2016), coinciding with important changes in neuroanatomy and brain structures, especially in neural circuits of the prefrontal region that are particularly susceptible to experiential input during this period of rapid growth and plasticity (Bick and Nelson, 2017).

### Preschool EFs and Later Academic Performance

As mentioned above, EFs are essential to the ability to perform academic tasks. Evidence for this claim has been obtained in samples of students of various ages, with and without learning difficulties, and with adequate and inadequate academic performance, independently of variables such as cultural context and socioeconomic level. EFs are so important in academic performance that they are even better predictors of academic performance than IQ (Viterbori et al., 2015; Purpura et al., 2017).

Several studies have shown that preschool EFs have an influence on students’ later skills in literacy and mathematics, the curricular areas in which the effect of EFs has been most studied.

#### Preschool EFs and Literacy Skills

In the area of literacy skills, verbal working memory is related to phonological awareness, which is necessary for the output of words and phrases—both spoken and written—and therefore in reading and writing. In order to produce a word or sentence, children must be able to hold multiple sounds or words in their memory and combine them (Purpura et al., 2017). Studies have found that dyslexic children, who often have phonological problems, perform more poorly on working memory tasks than typically developing children (Varvara et al., 2014). Reading comprehension is another literacy skill in which working memory plays a major role. When we read, we must relate the ideas that appear in each sentence and paragraph with those we have just read in the previous sentences and paragraphs. These ideas must be stored and activated in our mind and combined in a new structure, forming a whole that gives meaning to the text. Essentially, as we read, working memory plays a key role in storing the intermediate and final products of our computations, allowing us to build and integrate the successive ideas we extract from the text (Cartwright, 2015; García-Madruga et al., 2016). Similarly, working memory is also required in the composition of written texts and phrases. But if, while reading a text, we encounter information that is irrelevant or not of interest to us—for example, when scanning a text for information on a particular subject—we must be able to inhibit, reject, and not be distracted by any information that does not meet our needs, keeping our attention on the information that is relevant to our goal (Cartwright, 2015; García-Madruga et al., 2016; Purpura et al., 2017). Thus, inhibition also plays a role in reading comprehension—and in verbal and written expression—in close interaction with working memory and, as explained below, cognitive flexibility. It is therefore clear that learning and academic tasks require the simultaneous participation of the various EF components (Colé et al., 2014; Cartwright, 2015; García-Madruga et al., 2016; Rapoport et al., 2016).

Various researchers have also shown the significant contribution of cognitive flexibility to literacy skills such as phonological and print awareness, word reading, and reading comprehension. Cognitive flexibility is needed to create cross-modal connections between spoken and written language and to access and integrate the different characteristics of print (phonology, morphology, syntax, semantics) during the process of word recognition. Cognitive flexibility also has a critical role in reading comprehension, as we need to process phonological codes in order to recognize the written words while also processing the meaning of the words (Colé et al., 2014; Cartwright, 2015). Cognitive flexibility is thus a key process for understanding specific reading comprehension difficulties (Engel de Abreu et al., 2014). Students with these difficulties are relatively good at decoding, so they sound like good readers, but they have problems with comprehension. They focus inflexibly on decoding processes (i.e., on word-level features of print) and pay only limited attention to meaning. They have difficulty shifting their focus to the text’s meaning or to simultaneously managing decoding and the construction of meaning (Colé et al., 2014; Cartwright, 2015).

#### Preschool EFs and Math Skills

Regarding the contribution of EFs to children’s math skills, a substantial body of evidence shows that working memory is critical for mathematical proficiency. For example, calculation relies on working memory processes because it involves storing temporary information—the numbers involved in the operation, partial results, and the amount to be carried—and performing mental operations on this information until the final result is obtained. Working memory is especially important when the problem is presented verbally rather than visually (Clark et al., 2013; Rapoport et al., 2016). Nevertheless, several authors, in comparing primary school children with high and low working memory, found a significant difference in calculation ability even if the arithmetic operations were presented in written format (Viterbori et al., 2015). Number comparison is another math skill that requires holding multiple pieces of information (the numerals) in mind and combining or manipulating them in order to compare their magnitudes and identify the smallest or largest. Working memory also plays a role in the acquisition of new arithmetic facts—for example, addition and multiplication tables—because the operation and answer need to be held in mind together in order to strengthen the relationship between them (Cragg and Gilmore, 2014; Purpura et al., 2017). The influence of working memory in complex components of mathematics, such as problem-solving, can be illustrated in various ways. For example, when solving a problem, we must select the relevant information and hold it in mind. Some evidence suggests that poor problem solvers remember less relevant information than good problem solvers. The role of working memory in solving mathematical problems is closely related to a student’s ability to access the right information (e.g., appropriate algorithms) from long-term memory (Viterbori et al., 2015; Purpura et al., 2017).

As for inhibition, it is important at younger ages to suppress less sophisticated strategies (e.g., counting on from the first addend) in order to use more sophisticated strategies (e.g., counting on from the larger addend). Inhibition is also necessary in order to suppress answers to related but incorrect number facts (for example, in response to 4 × 4, children must inhibit 8, the solution to 4 + 4). Cross-operation errors such as these are generated by difficulty in inhibiting the incorrect responses in a set of possible and competing responses activated by the memory (Cragg and Gilmore, 2014; Viterbori et al., 2015). When a child is learning new concepts, inhibition—along with cognitive flexibility or shifting—is important in suppressing an automatic procedural approach and shifting attention toward the numerical relationships involved (Cragg and Gilmore, 2014). Inhibition also contributes to solving math problems, especially when the text of the problem contains irrelevant and distracting data that the child must suppress in order to develop an appropriate mental problem-solving model (Viterbori et al., 2015). Some studies show that students with mathematical difficulties have trouble inhibiting irrelevant information and focusing on relevant information (Cragg and Gilmore, 2014; Viterbori et al., 2015). However, when solving complex mathematical problems, children must also have cognitive flexibility in order to switch between different procedures (e.g., adding and subtracting) or to look for an alternative problem-solving procedure after attempting to solve a problem using an unsuitable procedure. Cognitive flexibility also appears to be related to more abstract aspects of mathematics, such as cardinal number knowledge (Purpura et al., 2017). When children progress from applying the counting sequence to sets (one-to-one correspondence) to achieving quantity (the cardinal number that represents a sum or total number of existing elements), they shift from thinking about counting as a procedure to thinking about it as a conceptual process. Specifically, children must adapt their thinking and flexibly move from the procedural task of counting to understanding counting as providing quantitative information (Viterbori et al., 2015; Purpura et al., 2017).

It is therefore clear that EFs make significant contributions to young learners’ overall mathematics and literacy performance.

### Systematic Observation in Preschool

The recent literature on early childhood education and development increasingly argues that the assessment of development processes and learning during preschool should be done primarily through systematic observation in the natural learning context (Early Head Start National Resource Center, 2013; Jablon et al., 2013). The literature also stresses that play—an activity inseparable from a child’s life—is an indispensable resource for the childhood teaching–learning process and for the systematic observation of children’s progress and development (Nell and Drew, 2013; Fasulo et al., 2017). Systematic observation of a child’s behavior during play makes it possible to obtain relevant data to describe, explain, and understand fundamental aspects of the child’s development and learning (Federici et al., 2017; Otsuka and Jay, 2017), including the development of EFs. Accordingly, the literature on EFs indicates that given children’s impulsive behaviors and linguistic, motor, and attentional limitations, the study of EF development in early childhood, like the tasks and tools used for their assessment, must be based on the children’s everyday activities (Nieto et al., 2016), such as play. However, few studies have used systematic observation of children’s play as a tool for obtaining objective and valid information about preschool EFs.

This lack of research may be due to certain difficulties associated with systematic observation, such as the high cost in terms of time (all observers must undergo rigorous prior training) and the painstaking process of collecting and recording the data (Portell et al., 2015). The time cost is even higher when the subjects observed are children, because of the additional complexity and difficulties inherent in working with young participants as a result of their developmental characteristics (behavioral instability, short attention span, and high fluctuation of motivation), the need to create a climate of trust to ensure the children’s well-being and participation, and legal and ethical requirements that must be met in order to comply with international research guidelines (Shaw et al., 2011). Because of the need to obtain informed consent from parents or guardians for children to participate in research, many studies involving children have small samples that are not very representative of the reference population. This could be a source of error and the results of such studies may not be generalizable to the reference population. However, new data analysis structures (such as G theory) are making it possible to overcome these limitations.

#### Generalizability Theory to Generalize Results from Systematic Observation of Preschool Behavior

In the field of education and development—and in the behavioral sciences generally—observed phenomena are often influenced by many factors, so the repetition of a particular experience or the use of a different instrument can modify the initial result considerably, leading one to wonder whether the observed values are interpretable or if they are the result of random fluctuations introduced by the act of measurement. This question is particularly important in behavioral observation designs. The use of G theory allows us to analyze the various sources of variance that can affect an observational measurement or measurement design and estimate the degree of generalization of a theoretical value with respect to specific conditions (Blanco-Villaseñor et al., 2014). However, G theory can be adapted to the specific conditions of each object of measurement, so its use in observational studies can contribute to the generalization of results and to improving their applicability on future occasions.

G theory assumes the existence of multiple sources of variance (variables or facets) in any measurement situation. This approach can estimate the accuracy of a measurement that is subject to multiple sources of error (Cardinet et al., 2010), allowing real variability to be separated from error variance. One of the important objectives of measurement is to try to identify and measure the components of variance that contribute to the error of an estimation and implement strategies that reduce the influence of these sources of error on the measurement.

As mentioned above, studies involving children often have a small sample size. On occasions, a “small” sample can be viewed as a possible limitation that could act as an additional source of measurement error. G theory allows us to analyze this source of variance and estimate the accuracy of the measurement in a studied sample. This makes it possible to estimate the degree to which the results obtained for a particular sample can be generalized to the reference population (Blanco-Villaseñor et al., 2014). Despite the advantages offered by this approach, few observational studies have used generalizability analysis, and even fewer have studied children. Fewer still have applied G theory to sample size estimation, given that G theory is normally used in observational studies to determine reliability and validity.

### Aims of the Present Empirical Investigation

Given the background set out above, the objectives of this study were as follows:

(1) To determine whether different EF levels measured in children through systematic observation at the end of preschool are associated with different levels of literacy and math skills the following year, that is, at the start of compulsory education.
(2) To determine whether the results obtained with the convenience sample recruited can be generalized to the reference population and, therefore, whether the studied sample is of sufficient size.

## Materials and Methods

### Ethics Statement

The study was carried out in accordance with the recommendations of the ethics committee at Zaragoza University and the principles of the Declaration of Helsinki. Written informed consent was obtained from the parents of all the children who participated. Each child received a small reward (two chocolates) for participating.

### Design

We used a multi-method design (Elliott, 2007; Sánchez-Algarra and Anguera, 2013; Kumschick et al., 2014; Mangelsdorf and Eid, 2015) consisting of systematic observation to observe preschool children during play and selective methodology to assess their reading, writing, and math skills the following year, that is, in the first year of compulsory primary education.

Systematic observation was non-participative and active and the behaviors observed were fully perceivable (Anguera, 2003; Shaughnessy et al., 2009; Bakeman and Quera, 2011).

The observational design was point, nomothetic, and multidimensional (Blanco-Villaseñor et al., 2003). It was point because a single session per participant was observed to assess each of the EFs analyzed; nomothetic because multiple observation units were analyzed; and multidimensional because several domains of EFs (working memory, inhibition, and mental flexibility) were analyzed within the theoretical model proposed by Miyake et al. (2000) and developed by other authors (e.g., Diamond, 2013).

### Participants

Forty-four Spanish participants were recruited. They were all students, aged 5–6 years, in their last year of preschool (last year of non-compulsory education in Spain) at the same school when the study started. The school was located in a central moderate-to-high income neighborhood of a Spanish city with approximately 700,000 inhabitants. The vast majority of the students approached (95.65% of all the children in their last year of preschool education) participated in the study. The other children (4.35%) did not participate as their parents did not provide their informed consent.

The students had to meet three inclusion criteria: (1) attendance at the targeted school since the second year of preschool education (age 3); (2) absence of the following disorders or risk factors: (a) birth weight <2000 g and/or gestational age <36 weeks or significant pre-, peri-, or postnatal events; (b) medical/neurological conditions affecting growth, development, or cognition (e.g., seizure) and sensory deficits (e.g., vision or hearing loss); (c) neurodevelopmental disorders (e.g., autism spectrum disorder, attention-deficit hyperactivity disorder, language disorder); (d) genetic conditions or syndromes; and (e) a first-degree relative with schizophrenia, bipolar disorder, or related disorders; and (3) an adequate IQ for their chronological age. The information to assess compliance with the first two criteria was provided by the children’s parents, and IQ was tested using the Spanish Battery of Differential and General Abilities Tests (BADyG) (Yuste and Yuste, 2001).

The sample was a convenience sample formed by children who met the inclusion criteria and whose parents signed the informed consent form authorizing their participation. **Table 1** summarizes the main descriptive characteristics of the sample.

**Table 1:** Descriptive characteristics of the sample.

*N*	*M*_Age_	Gender		*M*_IQ_	*M*_Birth weight_	*M*_Gestational age_
	(years) ± SD	♂	♀	(IQ ± SD)	(kg) ± SD	(weeks) ± SD
44	5.73 ± 0.30	16	28	90.05 ± 8.5	3.15 ± 0.47	39.06 ± 1.91

### Games

In order to obtain videos of the preschool children during play, each participant was offered the chance to participate in five games. These games were based on other non-standardized games and tasks that had been used in various studies to assess preschool EFs (Anderson and Reidy, 2012). Through the observation of the children’s spontaneous behavior in these games, it was possible to extract information about their EFs.

All of the games proposed to the children formed part of a fantasy story (the creation of a fantasy world is a characteristic of many children’s games; Garris et al., 2002). This fantasy story—in which each participant acted as the protagonist—was set in space, a topic that the teachers had indicated was of interest to the participating children. Although instructions were given for each game as part of the fantasy story, at no time were the child’s actions restricted or penalized in any way. Thus, the child was allowed to act freely throughout the course of the games.

#### Game 1: Preparing for the Journey

This game, based on the Backward Word Span task (Carlson, 2005; Diamond, 2013; Visu-Petra et al., 2014; Howard et al., 2015; Nieto et al., 2016), was used to observe behaviors indicative of the child’s verbal working memory. To explain the game to the child, the adult told the following story: “We’re going to take a trip to space in a big rocket ship. We need to prepare everything we’ll need for our trip. I’m going to say the names of several of these things, and I want you to repeat them back to me in reverse order. I’ll do two examples to help you understand better, and then you’ll continue. Okay?” The words used (e.g., milk, water) were familiar concepts to children of this age and consistent with their level of vocabulary development.

#### Game 2: Our Travel Companions

This game, based on the Backward Animal Images Span task (Diamond, 2013), was used to observe behaviors indicative of the child’s visuospatial working memory. To explain the game to the child, the adult told the following story: “Now we’re going to meet our travel companions. I’m going to show you some photos of them. Take a good look because I’m going to set the photos on the table, and then I’ll take them away. Then you’ll have to arrange the photos in the opposite order from how I put them on the table. I’ll do two examples to help you understand better, and then you’ll continue. Okay?” The images all showed common animals that preschool children learn about in class (e.g., dog, pig).

#### Game 3: The Flight Begins

This game, based on a traditional imitation game called Simon Says (Strommen, 1973), was used to assess behavioral inhibition. To explain the game to the child, the adult told the following story: “Now we’re flying in space! I’m going to indicate some actions and you have to do them. For example: If I say to touch your nose”—the adult performed this action while indicating it verbally—“you touch your nose.” The child was then given time to perform the action. The adult then continued explaining the game: “Now I’m going to say some more actions, but only do them if I first say ‘Simon Says’. If I don’t say ‘Simon Says’ before indicating the action, don’t do it; just hold still.” The adult ordered an action while performing it simultaneously, but without first saying “Simon Says,” leaving time for the child to remain still. In this game, therefore, in the absence of the words “Simon Says,” the child was expected to be able to refrain from performing the action despite being told to and despite seeing the adult do it.

#### Game 4: The Day-Night Planet

This game, based on the Day-Night Task (Gerstadt et al., 1994; Carlson, 2005), was used to observe behaviors indicative of the child’s capacity for resistance to distractor interference. To explain the game to the child, the adult told the following story: “We’ve landed on a new planet! On this planet, when you see the sun”—a picture of a sun appeared on a computer screen—“it’s nighttime. When you see the moon”—a picture of a moon appeared on a computer screen—“it’s daytime. The sun and the moon are going to appear on the screen quickly, one at a time. Pay attention, because when you see the sun”—the picture of the sun once again appeared on the screen—“you have to say ‘night’ as fast as you can, and when you see the moon”—the picture of the moon once again appeared on the screen—“you have to say ‘day’ as fast as you can.” Two images of the sun and two images of the moon were shown alternately, as an example, to ensure that the participant had understood the instructions.

#### Game 5: Martians

This game is based on the Shape School game, which was created by Espy (1997) to assess cognitive flexibility and resistance to distractor interference in preschool children. To explain the game to the child, the adult told the following story: “Let’s meet the inhabitants of this new planet!”—the adult showed the child a piece of cardboard with red, blue, and yellow squares and circles representing neutral facial expressions—“Look. These are the Martians who live on this planet. Their name is their color. Tell me the names of all the inhabitants of this planet as quickly as you can.” The adult then displayed another piece of cardboard showing Martians with happy and sad faces. The adult said to the child: “Now some of the Martians are sad because they have to go home. Tell me, as quickly as possible, the name of the Martians with a happy expression but not the name of those with a frustrated face.” This allowed the observation of behaviors related to resistance to distractor interference (i.e., resisting the sad faces and therefore not saying their color). Afterward, the adult displayed a third piece of cardboard showing some of the previous Martians, as well as some new Martians wearing hats. All of the Martians had a neutral face. The adult said to the child: “New Martians have arrived! These new Martians are wearing a hat, and their name is the shape of their figure. Take a good look and tell me the names of all the Martians as quickly as possible. Remember that the name of the Martians who aren’t wearing a hat is their color and the name of the Martians wearing a hat is their shape.” This allowed the observation of behaviors related to cognitive flexibility. Later, the adult displayed a fourth piece of cardboard showing both types of Martians (with and without a hat) with happy or frustrated faces. The adult said to the child: “Now there are Martians with a happy expression and others with a frustrated face. As quickly as possible, say the name of the happy Martians. Remember that the name of the Martians without a hat is their color and the name of the Martians with a hat is their shape.” This allowed the observation of behaviors indicative of the child’s capacity for resistance to distractor interference (as the child had to refrain from naming Martians with frustrated faces) and cognitive flexibility (as the child had to switch between shape and color to name the happy Martians depending on whether or not the Martian was wearing a hat).

### Instruments for Collecting Data through Systematic Observation

In systematic observation (Anguera, 2003; Sánchez-Algarra and Anguera, 2013; Arias-Pujol and Anguera, 2017), a distinction is made between recording instruments (i.e., those used to record or code data) and observation instruments (purpose-designed instruments to analyze a given subject).

#### Recording Instruments

A Sony HDR-CX115 video camera was used to record the activity of each preschool child during the games.

The open-source software application Lince (Gabin et al., 2012) was used to code actions indicative of the preschool children’s EFs. This program can be downloaded for free from http://lom.observesport.com/. Lince can be used to code all types of behavior as it is the observer who imports the video recordings and corresponding observation instrument into the program. The program allows observers to simultaneously view the video recordings, the observation instrument, and the dataset being created.

#### Observation Instrument

As required by the nature of our systematic observation design, we built an *ad hoc* instrument fully adapted to the context of interest to capture the children’s level of EF, using games (tasks) performed by the children. As the design was multidimensional, we built an instrument combining a field format and category systems (Sánchez-Algarra and Anguera, 2013; Castañer et al., 2016). The instrument had seven dimensions, each of which formed the basis for a category system of exhaustive and mutually exclusive categories. The seven dimensions corresponded to three types of criteria: three fixed criteria, which remained unchanged throughout the observation session; one mixed criterion, which remained unchanged for part of the session; and three variable criteria, which changed frequently throughout the sessions and corresponded to the behaviors that were observed and coded. The observation instrument is shown in **Table 2**.

**Table 2:** Observation instrument.

Criterion	Dimension	Category systems	Category code	Category description
Fixed	Participant	Participant	01	Participant 1 is performing the task and is being assessed
			02	Participant 2 is performing the task and is being assessed
			03	Participant 3 is performing the task and is being assessed
			04	Participant 4 is performing the task and is being assessed
			…	Participant … is performing the task and is being assessed
	Gender	Female	F	The participant performing the task is female
		Male	M	The participant performing the task is male
	Executive Function Task	Backward Word Span	BWS	Task for assessing verbal working memory
		Backward Animal Images Span:	BAIS	Task for assessing visuospatial working memory
		Simon Says	S	Task for assessing behavioral inhibition
		Day-Night Task	DNT	Task for assessing resistance to distractor interference
		Shape School	SS	Task for assessing cognitive flexibility primarily but also inhibition
Mixed	Phase	Phase 1	P1	Phase 1 of the task the participant is performing
		Phase 2	P2	Phase 2 of the task the participant is performing (only possible in the Shape School tasks)
		Phase 3	P3	Phase 3 of the task the participant is performing (only possible in the Shape School task)
		Phase 4	P4	Phase 4 of the task the participant is performing (only possible in the Shape School task)
Variable	Item	Item 1	1	Response stimulus 1 in task
		Item 2	2	Response stimulus 2 in task
		Item 3	3	Response stimulus 3 in task
		Item 4	4	Response stimulus 4 in task
		Item 5	5	Response stimulus 5 in task
		Item 6	6	Response stimulus 6 in task
		Item 7	7	Response stimulus 7 in task
		Item 8	8	Response stimulus 8 in task
		…	…	
		Item 40	40	Response stimulus 40 in task
	Performance	Correct	Co	The participant performs adequately for the specific task item
		Incorrect	Inc.	The participant does not perform adequately for the specific task item
		Self-Correct	Aut	The participant starts an action, realizes and corrects a mistake, and does not complete the action
		Omission	Om	The participant does not respond when required to do so by the specific task
	Adult	Explains	AdEx	The adult explains to the participant what the task consists of by giving instructions and explaining the rules of the game
		Corrects	AdCg	The adult corrects the participant when he/she does something wrong, leading the participant to either correct the mistake and or to modify his/her approach accordingly

### Standard Instruments

The two standard instruments used in this study justify the incorporation of selective methodology in the systematic observation and, consequently, a multimethod approach.

#### BADyG: Assessment of Intellectual Ability

The BADyG (Yuste and Yuste, 2001) was used to assess intellectual ability and confirm that the children had an adequate IQ for their chronological age (third inclusion criterion). The BADyG is a Spanish battery of nine tests that have proven to provide a reliable measure (high Cronbach’s alpha values) of the intellectual abilities of school children in numerous studies (Castejón et al., 2016; Veas et al., 2016). In our study, we used the level-1 battery designed for use in preschool children (BADyG-I).

The BADyG-I assesses three global performance items: (1) Verbal Intelligence, assessed through Numerical-Quantitative Concepts (1a), Information (1b), and Graphic Vocabulary (1c); (2) Non-verbal Intelligence, assessed through Non-verbal Mental Ability (2a), Reasoning with Figures (2b), and Logic Puzzles (2c); and (3) General Intelligence and IQ, assessed using the scores from the previous tests. Each test is composed of 18 items, each consisting of five pictures. The students must mark with an X the picture that matches the statement read out by the test administrator.

The children were also administered the complementary Perception and Coordination Graphomotor skills test to assess their ability to coordinate vision and manual movements during the reproduction of 12 simple geometric figures.

#### PAIB 1: Assessment of Academic Skills

The PAIB 1 (*Prueba de aspectos instrumentales básicos: Lectura, escritura y conceptos numéricos*; Galve-Manzano et al., 2009) was used to assess academic skills in reading, writing, and numeracy. These skills are considered to be the most important pillars for academic success (Cutler and Graham, 2008).

The PAIB 1 consists of eight subtests with activities that the children must complete with a pencil and paper. The activities are structured to resemble typical classroom activities. The PAIB 1 has demonstrated reliability (Galve-Manzano et al., 2009).

The eight subtests are:

1. *Basic Aspects of Mathematics* (three tests):
1.1. *Numeracy.* A 10-item test involving activities related to numbers with which the children are familiar.
1.2. *Calculation.* Six simple arithmetic operations.
1.3. *Problem resolution.* Eight math problems.
2. *Basic Aspects of Reading and Writing* (five tests):
2.1 *Reading comprehension.* Seven-item reading comprehension test consisting of sentences with different levels of grammatical complexity. Each item consists of four true or false sentences about a drawing. The children must choose the true sentences.
2.2 *Writing.* Subtest designed to assess writing skills through four tests or tasks involving different cognitive processes.
2.2.1. *Spelling.* This subtest consists of two tests: (a) *Word dictation*, where the children must write down words with different syllable structures read out by the test administrator, and (b) *Sentence dictation*, where the children must write down four sentences read out by the administrator.
2.2.2. *Vocabulary*. This subtest also consists of two tests: (a) *Sentence composition (I)*, in which the children must write a sentence using all of three words provided, and (b) *Sentence composition (II)*, where the children are shown four drawings and asked to think about what is happening and to write a sentence for each of them.

A score is calculated for each of the eight tests, together with a total score for math, a total score for reading and writing, and a total score for math, reading, and writing combined.

### Data Analysis Software

Ensuring the quality of the data collected is an essential part of systematic observation. We assessed this by calculating intra- and interobserver reliability for 30 sessions using the intraclass correlation coefficient in SAS 9.1.3 (Schlotzhauer and Littell, 1997; SAS Institute Inc., 2004).

The data used to address the first study objective were analyzed in the general linear model (GLM) in SAS 9.1.3 (Schlotzhauer and Littell, 1997; SAS Institute Inc., 2004).

The generalizability analysis to assess sample size (second study objective) was performed in EduG 6.0-e (Cardinet et al., 2010).

### Procedure

The study was approved by the school management team and the parents of the children in the last year of preschool education were informed about the goals and nature of the study. They were asked to consent to their children participating in the study and to give their permission to have them video recorded while playing. They were also asked questions to assess compliance with the first two inclusion criteria: (1) attendance at the school since the second year of preschool education (age 3) and (2) absence of certain disorders or risk factors. Anonymity and compliance with ethical principles were guaranteed.

Students for whom parents gave their informed consent to participate in the study and who met the first two inclusion criteria were tested for IQ to ensure that they also met the third criterion, which was an adequate IQ for their chronological age. This was tested using the BADyG-I, which was administered to the group as a whole in two sessions held on non-consecutive days. Each session lasted approximately 30 min. The tests were administered according to the instructions in the BADyG-I manual for children in preschool education. They were administered by the same person, with the help of three others. They were scored automatically using the computer software feature provided with the BADyG-I. Each child was scored on verbal intelligence, non-verbal intelligence, and general intelligence. All the students had an adequate IQ for their chronological age and were therefore admitted into the study.

To fulfill the requirements of systematic observation, several exploratory play sessions were held prior to the definitive systematic observation. A child and the researcher were present in each session. These sessions were held at the school, but not in the children’s usual classroom to avoid distractions. They were held during school hours and the children were allowed to take their usual breaks. The aim of this exploratory phase was to guarantee the consistency of subsequent decisions and collect information to guide the construction of the observation instrument (Anguera, 2003). Specifically, the exploratory sessions were intended to verify that the children understood and were interested in the games, thus ensuring that they would participate readily and naturally. The children’s involvement in the games is what would make it possible to systematically observe actions indicative of their EFs. The exploratory sessions also allowed the researchers to determine the approximate length of time that the children would spend on the games. On this basis, the researchers were able to determine how many sessions would be needed in order for each participant to play all the games. These steps were taken in order to ensure that the games could be included in the children’s regular play routines without altering their activities or the context. Each day, the students’ regular preschool schedule included periods of playtime as well as other regular activities that are common in school settings (psychomotor activities, reading and writing, lunch, rest periods, etc.). Thus, the exploratory sessions consisted of three children playing, on an individual basis, each of the five games described above after receiving the aforementioned explanations. The first child played all five games in a row, in a single session and in the following order: Games 1, 2, 3, 4, and 5. The session lasted 32 min, longer than the usual time allocated for play in the children’s school routines. As a result, to avoid altering the children’s daily school activities, we decided to offer the second child the chance to play the games in two sessions on different days. Thus, Games 1, 2, and 3 (involving the trip to space and the preparation thereof) were offered during the first session and Games 4 and 5 (set on the destination planet) were offered during the second session. The first session lasted 17.45 min and the second session lasted 8.20 min, and therefore was in line with the usual amount of playtime in the children’s school routine. The same approach was used for the third child. The first session lasted 15.30 min and the second session lasted 7.10 min, thus respecting the usual amount of playtime in the daily school routine.

On the basis of this exploratory analysis involving three children who played the five games individually and freely, the following decisions were made:

(a) The games were deemed to be useful and appropriate for the systematic observation of preschool EFs, as they consisted of games that the children understood and found interesting. The children expressed their satisfaction and enjoyment of the games and exhibited spontaneous play activity.
(b) Each participant would be observed during two play sessions on different days. Each session would have a maximum duration of 20 min. With this arrangement, the students’ usual play routines would be respected and their school activities would remain unchanged.
(c) Each participant would play Games 1, 2, and 3 in the first session and Games 4 and 5 in the second session.

The sessions were video recorded for later viewing. The recordings were used to integrate information about the children’s EFs during completion of the different games and information from the theoretical framework on EFs in children with the ultimate aim of building the observation instrument. Different versions of the instrument were built and improved on until the definitive version shown in **Table 2** was achieved.

In the definitive systematic observation stage, each participant completed all the EF games. This was done at the school, again outside the children’s classrooms and without interference from their teacher or other students. The games were played on two separate days. On the first day, the children played Game 1 (Preparing for the Journey), Game 2 (Our Travel Companions), and Game 3 (The Flight Begins) in a single session. The mean time spent on these games was 16.33 min. A week later, they played Game 4 (The Day-Night Planet) and Game 5 (Martians), again in a single session. The mean time spent on these games was 9.31 min. None of the sessions exceeded the length of the children’s usual playtime; thus, their daily school routines were maintained. All the sessions were video recorded.

The video recordings were imported into Lince and coded using the *ad hoc* observation instrument for assessing EF (**Table 2**). The data recorded were converted into a matrix of codes that was tested for reliability (intraclass correlation coefficient ≥0.95).

The following year, when the children were in their first year of compulsory education, they were administered the PAIB 1 to test their reading, writing, and math skills. They completed the test as a group, in two sessions on non-consecutive days, and it was administered by the same adults who had administered the BADyG-I the previous year following the instructions in the manual. The first session lasted approximately 45 min and the second session was slightly shorter, at 40 min. The tests were corrected automatically by computer and a score was given for each of the eight tests, together with a total score for math, a total score for reading and writing, and a total score for these combined.

### Data Analysis

GLMs were used to analyze the data to address the first study objective, which was to investigate whether different levels of EF in preschool children were associated with different levels of reading, writing, and mathematical skills the following year, at the start of compulsory education. GLMs indicate the percentage of variance in the dependent (response) variable (in our case, level of academic skills) that is explained by a series of independent (explanatory) variables (in our case, EF level and other variables that we will specify further on).

In order to estimate these models, it was first necessary to transform the data corresponding to the categories in the Performance dimension in the observation instrument into an appropriate format. To do this, we first transformed the data corresponding to the execution of each game into raw scores, assigning 2 points to the Correct category, 1 point to the Self-Correct category, and 0 points to the Incorrect and Omission categories. This resulted in a raw score per participant per EF game completed (EF level).

The converted data were now suitable for fitting various GLMs in SAS. Academic skills level was used as the response variable in all the models. The explanatory variables were EF level in all cases and, depending on the model, participants, gender, EF game, and academic skills assessment instrument, together with their different interactions. The coefficient of determination (*R*^2^) was calculated for all models. This coefficient (expressed as a percentage) indicates the extent to which the model (with its explanatory variables) explains the variance in the response variable (reading, writing, and mathematical skills).

To address the second objective of the study, i.e., to determine whether our systematic observations were generalizable to the reference population from which the sample was drawn, we calculated the generalizability coefficient using the G theory software program EduG. We used a measurement design with EF level and academic skills instrument as the differentiation facets and participants as the instrumentation facet.

## Results

**Tables 3** and **4** show the most relevant results for the primary study objective, which consisted of estimating a GLM that would provide the best explanation for the variance in literacy and math skills.

**Table 3:** Two type 1 overall general linear models, one with three variables and another with five.

Source	df	SS	MS	*F*-value	Pr > *F*
	*Three facets with interactions (EF level, participants, academic skills instrument)*				
Model	1264	956167.91	756.46	234.71	<0.0001
Error	1617	5211.43	3.22		
Total corrected	2881	961379.34			
*R*^2^					0.9946
	*Five facets with interactions (EF level, EF games, participants, academic skills instrument, and gender)*				
Model	281	896834.70	3191.58	128.56	<0.0001
Error	2600	64544.64	24.82		
Total corrected	2881	961379.34			
*R*^2^					0.9329

**Table 4:** Two type 1 specific general linear models, one with three variables and another with five.

Source	df	SS	MS	*F*-value	Pr > *F*
	*Three facets: EF level, participants, and academic skills instrument with their relevant interactions*				
EF level	2	1269.17	634.58	196.90	<0.0001
Participants	43	46290.20	1076.52	334.02	<0.0001
EF level × participants	69	1535.20	22.25	6.90	<0.0001
Academic skills instrument	10	837107.72	83710.77	25973.7	<0.0001
EF level × academic skills instrument	20	1917.42	95.87	29.75	<0.0001
Participants × academic skills instrument	430	66537.60	154.74	48.01	<0.0001
EF level × participants × academic skills instrument	690	1510.59	2.19	0.68	1.0000
	*Five facets: EF level, EF games, participants, academic skills instrument, and gender with their relevant interactions*				
EF games	4	87.18	17.44	0.70	0.6217
EF level	2	1277.52	638.76	25.73	<0.0001
EF games × EF level	10	1518.12	151.81	6.12	<0.0001
Participants	43	44832.11	1042.61	42.00	<0.0001
EF games × participants	201	3763.32	18.72	0.75	0.9952
Academic skills instrument	10	837973.09	83797.31	3375.54	<0.0001
Gender × academic skills instrument	10	7383.36	738.34	29.74	<0.0001

**Table 3** shows the two models that provided the best fit. The first had three explanatory variables (EF level, participants, and academic skills instrument, together with their interactions), while the second had five explanatory variables (EF level, EF games, participants, academic skills instrument, and gender, also with their respective interactions). In both cases, there were significant differences, indicating that level of academic skills in the first year of compulsory education was explained by three variables in the first model and five in the second one. The three-variable model accounted for 99% of the variance (*R*^2^ = 0.99), while the five-variable model accounted for 93% (*R*^2^ = 0.93). Both models, therefore, provided a very good fit, although the three-variable model slightly outperformed the five-variable one. The results suggest that the additional variables in the second model (EF games and gender) did not contribute anything to the overall model. On the contrary, they appeared to somehow distort it as it explained less of the variance.

The variables in the three-variable model (EF level, participants, and academic skills instrument) explained almost all of the variance in reading, writing, and math, and their power was not improved by the addition of more variables.

**Table 4** shows the results for the three- and five-variable models, including the individual components of variance and their relevant interactions. Interactions that did not make a significant contribution have been omitted. In the three-variable model in **Table 4**, all the components and their interactions showed significant differences, except for the largest order interaction component EF level × participant × academic skills instrument (residual error of the model). In brief, EF level, participants, and academic skills instrument contributed significantly to explaining 99% of variations in literacy and math skills in the first year of compulsory education. The 1% of unexplained variance suggests the existence of variance components that were not included in our study. This is supported by the fact that when we included other variables contemplated in our analysis (e.g., in the five-variable model), these not only reduced the fit of the model, but also, in some cases, offered no significant differences (**Table 4**), indicating that they did not explain variations in academic skills as they contributed nothing to the overall model. This was the case, for example, for EF game (0.6217) and EF game × participants (0.9952). A similar situation was seen for gender and a number of its interactions (e.g., gender × EF level), which were eliminated from **Table 4** as they did not make any relevant contribution to explaining the variance in academic skills. Significant differences were, however, obtained for gender × academic skills instrument, and for EF level, EF games × EF level, participants, and academic skills instrument, meaning that they also contributed to explaining variability.

The results for the generalizability analysis are summarized in **Tables 5** and **6**. **Table 5** shows the estimated variance components. The academic skills instrument has a large influence on the facet, accounting for 88.2% of all variance in the three-facet design. As can be seen, the rest of the facet and its interactions contributed very little to design variability.

**Table 5:** Analysis and G study (generalizability theory) to estimate the generalizability of the results obtained for the sample of participants using the three-facet design, EF level (L) × participants (P) × academic skills instrument (S).

				Components				
Source	SS	df	MS	Random	Mixed	Corrected	%	SE
L	1269.00	2	634.50	1.08	1.08	1.08	0.2	0.93
P	46290.00	43	1076.51	27.44	27.44	27.44	3.8	6.89
S	837107.00	10	83710.70	632.29	632.29	632.29	88.2	258.90
LP	1535.00	86	17.85	1.46	1.46	1.46	0.2	0.24
LS	1917.00	20	95.85	2.14	2.14	2.14	0.3	0.66
PS	66537.00	430	154.74	50.99	50.99	50.99	7.1	3.51
LPS	1510.00	860	1.76	1.76	1.76	1.76	0.2	0.08
Total	956165.00	1451					100	

**Table 6:** G study (generalizability theory) to estimate the generalizability of the results obtained for the sample of participants using the three-facet measurement design, EF level (L) × participants (P) × academic skills instrument (S).

Source of variance	Differentiation variance	Source of variance	Relative error variance	% Relative	Absolute error variance	% Absolute
L	1.08		.....		.....	
	.....	P	.....		0.62	33.6
S	632.29		.....		.....	
	.....	LP	0.03	2.7	0.03	1.8
LS	2.14		.....		.....	
	.....	PS	1.16	94.1	1.16	62.4
	.....	LPS	0.04	3.2	0.04	2.2

Sum of variances	635.51		1.23	100%	1.86	100%
Standard deviation	25.21		Relative SE: 1.11		Absolute SE: 1.36	

Coef_G relative ξρ^2^(δ)	1.00
Coef_G absolute ξρ^2^(Δ)	1.00

**Table 6** summarizes the results of the G study. The generalizability coefficient [ξρ^2^(δ) = 1] indicates that the sample size (44 participants) was sufficient for accurately generalizing the results to the larger universe from which the sample was obtained.

## Discussion

The results of this study show that preschool EF level together with participants and academic skills instrument explained 99% of variations detected in literacy and math skills of children in their first year of compulsory education. In addition, our findings appear to be highly generalizable to the reference population from which the sample was drawn.

Overall, our results are consistent with reports in the literature that EFs have a key role in reading, writing, and math skills and that early assessment of these functions can help to identify children who are likely to present later learning difficulties (Engel de Abreu et al., 2014; McClelland et al., 2014; Viterbori et al., 2015; Moriguchi et al., 2016; Nieto et al., 2016; Purpura et al., 2017).

The interaction gender × EF level was not significant in our results, indicating the absence of significant differences in EF between boys and girls. While our systematic observation that EFs develop at a similar rate in boys and girls finds some support in the literature (Anderson, 2002; Li et al., 2009), several studies have reported that girls have slightly higher EF abilities than boys (Clark et al., 2013; Mansouri et al., 2016), at least in the case of certain components, such as inhibition (Mansouri et al., 2016). Nonetheless, there have also been reports of boys outperforming girls in components such as working memory (Dias et al., 2013). Differences in EF abilities between the sexes have been attributed to biological differences in frontal and temporal lobe function in children. They would thus be attributable to different brain growth patterns, which appear to follow the prefrontal cortex connections involved in the different EF components (Ngun et al., 2011). However, this is an area that requires further research.

The development of EFs is the result of interactions between biological growth factors and individual experiences, suggesting that they are malleable and as such candidates for targeted interventions (Diamond, 2013; Traverso et al., 2015; García-Madruga et al., 2016). The results of our study have important implications for educational practice. Assessment of EFs in preschool children may identify children whose EF level is lower than expected for their age and could therefore present later learning difficulties, enabling thus early interventions aimed at optimizing EFs with the ultimate goal of improving essential academic skills, such as reading, writing, and math. A growing number of interventional strategies are proving to be effective in this respect and many revolve around everyday activities, meaning they do not require a costly infrastructure (Anderson and Reidy, 2012; Diamond, 2014; Zelazo et al., 2016).

Children who start school with delays or gaps in the skills or EFs required for learning have been seen to continue to have difficulties throughout school, and the gaps tend to increase as the children move up through the school system (Clark et al., 2013). Therefore, early assessment of EFs, followed by early intervention when necessary, should be implemented as an educational action in all school systems. In addition, the benefits of early interventions persist into later life. In short, early interventions targeting EFs can benefit children’s cognitive, social, and emotional development, but they can also benefit development in later years, contributing to personal and career success, health, and quality of life in general (Diamond, 2013; Howard et al., 2015; Moriguchi et al., 2016). There is abundant literature showing that effective investment in early childhood education has a greater impact than later interventions and that the effects persist beyond the duration of the intervention, benefiting thus not only individuals but society as a whole. A country’s socioeconomic progress and the well-being of its citizens are closely linked to academic achievements, which, in turn, are associated with adequate EF development in the early years of life. The benefits of early intervention in EFs thus far outweigh their potential costs, as the return on investment brings benefits to both children and the nation (Duncan and Magnuson, 2013; Bartik, 2014; Kaufman et al., 2015).

As children get older, they are presented with increasingly demanding tasks and academic challenges, but their EFs also improve. The improvement, however, is irregular (i.e., it is characterized by cycles of jumps and drops), so the relationship between these variables could show variations with age (Cragg and Gilmore, 2014; Viterbori et al., 2015; Moriguchi et al., 2016). It would also be interesting to investigate this aspect of EF further.

This study has contributed to knowledge in the area of preschool EFs and to systematic observation, as it demonstrates once again that the systematic observation of behavior in natural settings is a particularly apt scientific method for studies in the areas of development and education. This method offers endless opportunities for expanding knowledge in these areas, particularly in young children.

Systematic observation aims to describe and explain phenomena that occur in natural settings (Anguera, 2003) and aside from home, there is no more natural setting for children than school. School has an obvious impact on a child’s development and life in general. Together with family, it is the factor that influences early development most (Bronfenbrenner, 1989). One of the greatest merits of systematic observation thus is that it captures development and learning almost as it occurs in everyday life, not in the controlled, artificial environment of a laboratory, enabling thus the rigorous analysis of everyday behavior in a person’s natural settings (Anguera, 2003; Early Head Start National Resource Center, 2013). The most recent literature on the development of EFs highlights both the need for and the benefits of assessing EFs while children are performing routine, everyday activities in familiar contexts, as this is where EFs are developed, not in the controlled structure of a laboratory (Willoughby et al., 2012; Nieto et al., 2016). Before these recommendations, EFs were typically assessed using tasks completed by children in clinical or laboratory settings or surveys or questionnaires on their behavior filled in by third parties, such as parents or teachers. Both systems have their limitations. Tasks completed in a laboratory-like setting will reflect how a child behaves in this artificial, controlled setting but not in the real world, and any findings thus will have low ecological validity (Miranda et al., 2016). In the second case, while third parties can provide information on how children behave in a greater variety of situations, the reliability of this information is questionable for numerous reasons. The answers might be biased by recall or a desire to answer what is “socially acceptable,” for example, or the person may be unfamiliar with or fail to perceive certain behaviors (Wertz, 2014). Systematic observation, by contrast, has high ecological validity as it captures spontaneous behavior in natural settings. It also has an additional advantage that the behaviors are observed and coded by one or more people who are experts not only in the “what” but also in the “how” (Anguera, 2003). Our study thus contributes to advancing research in early EFs, as it was conducted in line with the latest guidelines for research in this area. We hope that more studies will take on board this recommendation to assess EFs in natural settings.

One issue of increasing concern to methodologists and researchers in field of education and development and in the social sciences in general is the quality of data gathered during the research phase, as this has an obvious impact on findings and subsequent decisions. The importance of reliable data is a given in all methodological approaches, but being able to offer the necessary guarantees of quality is particularly challenging when studying spontaneous behavior in natural settings. When perceivable human behavior is observed without the constraints imposed by external controls, the data collected are more likely to contain more errors and more serious errors, potentially jeopardizing the validity of the research.

One means of addressing the different risks that can affect the accuracy of a dataset is to design a quality control procedure that analyzes how different facets or potential sources of variance affect different measurements designs and also provides a measure of the magnitude of error. The relatively recent use of G theory to calculate the reliability and validity of observational data is an important step in this direction, as is its lesser known application for estimating effective sample size. We used this novel feature of G theory to avoid underpowering, which is a frequent limitation of studies conducted in children.

## Author Contributions

EE-P contributed to conceptual structure, collecting data, and systematic observation. MH-N involved in collecting data. AB-V performed data analysis and results. MTA contributed to conceptual structure and systematic observation. All authors contributed to documenting, drafting and writing the manuscript, and gave their approval to the final version to be published.

## Conflict of Interest Statement

The authors declare that the research was conducted in the absence of any commercial or financial relationships that could be construed as a potential conflict of interest. The reviewer EP and handling Editor declared their shared affiliation.
